# Circular RNA Foxo3: A Promising Cancer-Associated Biomarker

**DOI:** 10.3389/fgene.2021.652995

**Published:** 2021-03-23

**Authors:** Tianli Yang, Yang Li, Feng Zhao, Liuhua Zhou, Ruipeng Jia

**Affiliations:** Department of Urology, Nanjing First Hospital, Nanjing Medical University, Nanjing, China

**Keywords:** FOXO3, cancers, microrna sponge, circular RNAs, biomarker

## Abstract

Circular RNAs (circRNAs) are a class of novel non-coding RNAs (ncRNAs). Emerging evidence demonstrates that circRNAs play crucial roles in many biological processes by regulating linear RNA transcription, downstream gene expression and protein or peptide translation. Meanwhile, recent studies have suggested that circRNAs have the potential to be oncogenic or anti-oncogenic and play vital regulatory roles in the initiation and progression of tumors. Circular RNA Forkhead box O3 (circ-Foxo3, hsa_circ_0006404) is encoded by the human FOXO3 gene and is one of the most studied circular RNAs acting as a sponge for potential microRNAs (miRNAs) (Du et al., 2016). Previous studies have reported that circ-Foxo3 is involved in the development and tumorigenesis of a variety of cancers (bladder, gastric, acute lymphocytic leukemia, glioma, etc.). In this review, we summarize the current studies concerning circ-Foxo3 deregulation and the correlative mechanism in various human cancers. We also point out the potential clinical applications of this circRNA as a biomarker for cancer diagnosis and prognosis.

## Introduction

Cancer has become the most severe public health problem, ranking as the leading cause of death worldwide. According to GLOBOCAN estimates in 2018, 18.1 million new cancer cases and 9.6 million cancer-related deaths occurred worldwide (Barrett and Salzman, 2016). Thus, a series of potential diagnostic and prognostic biomarkers urgently need to be explored.

Circular RNAs (circRNAs) are an unusual class of RNA molecules with a circular structure formed by a back-splicing mechanism that joins the 3′ and 5′ methylguanosine caps together (Guerra et al., 2020). Although this special molecular characteristic limits the normal transcription of circRNAs, an increasing number of circRNAs have been identified with high-throughput sequencing technology (Barrett and Salzman, 2016; Li X. et al., 2018). CircRNAs can specifically combine with microRNAs (miRNAs) as competitive endogenous RNAs (ceRNAs) to regulate the expression of relevant genes by acting as miRNA sponges. The special structure that prevents exonuclease-mediated degradation makes circRNAs very stable (Jeck and Sharpless, 2014). In addition, circRNAs can interact with proteins as transcriptional regulators and translation templates. For examples, ciRS-7 is a classic circRNA that acts as a “supersponge” and highly expressed in neuronal tissues. Piwecka et al. (2017) found that ciRS-7 knockout mice tended to develop neuropsychiatric disorders, and the mechanism was related to the ciRS-7/miR-7/Fos axis. Another study showed that exonic-intronic circRNAs (EIciRNAs) enhanced the expression of their cis-genes and promoted transcription via EIciRNA-U1-snRNP complexes (Li et al., 2015).

The forkhead box O subclass of transcription factors mainly includes Foxo1, Foxo3, Foxo4, and Foxo6. Foxo genes were initially identified as insulin-associated genes; however, emerging studies have proven that they are actually involved in many biological process, such as cell metabolism, cell proliferation, cell survival and apoptosis (Shatseva et al., 2011; Stefanetti et al., 2018). Foxo3 is closely related to longevity through the phosphatidylinositol 3-kinase (PI3K)/Akt pathway (Manning and Toker, 2017). Circ-Foxo3 is highly expressed in normal tissues, while the downregulation of circ-Foxo3 usually occurs in the development of cancer (Cho et al., 2014). This review mainly summarizes the recent knowledge about the characteristics of circ-Foxo3 in the biological behaviors and molecular mechanisms of human cancers. Moreover, the potential of circ-Foxo3 as a prognostic biomarker and therapeutic target is also debated.

## Biogenesis and Classification

Different from linear RNA, circRNA is characterized by a covalent closed-loop structure which protects circRNA from the degradation of RNases. With the widespread application of high-throughput sequencing and quantitative PCR, most circRNAs that are generated from precursor mRNAs (pre-mRNAs) via back-splicing or lariat circulation have attracted the attention of scholars (Szabo and Salzman, 2016; Li X. et al., 2018). According to recent studies, circRNAs can originate from various regions, such as exons, introns, intergenic regions, and antonymous and untranslated regions. Different splicing and circularizing mechanisms lead to four major types of circRNAs, namely, exonic circRNAs (ecircRNAs); EIciRNAs; circular intronic RNAs (ciRNAs), which exist mainly in the nucleus; and transfer RNA (tRNA) intronic circRNAs (tricRNAs), which originate from tRNA introns via pre-tRNA splicing enzymes; ecircRNAs account for the largest proportion of circRNAs (Barrett and Salzman, 2016; Huang G. et al., 2017).

## Functions

### Effect of miRNA Sponges

**FIGURE 1:**
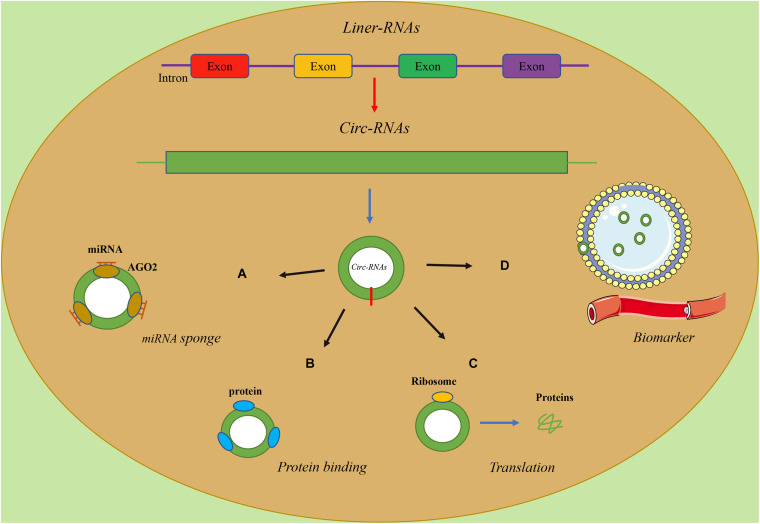
The functions of circRNAs.

Theoretically, any type of RNA with miRNA response elements (MREs) can bind to miRNAs and thus function as a ceRNA. Studies have found that circRNAs have MREs, so they naturally have the ability of sponge miRNAs (Figure 1A) (Hansen et al., 2013). Consequently, the sponging effect of circRNAs reduces the miRNA-mediated suppression of downstream gene expression. CircRNAs are involved in diverse tumorigenic processes via these molecular mechanisms, while the majority of circRNAs are maintained at a low level under normal conditions (Guo et al., 2014). For instance, Zhang et al. (2019) found that circ-Foxo3 and miR-138-5p/miR-432-5p were enriched for AGO2 through an anti-AGO2 RIP assay, indicating that circ-Foxo3 downregulated NFAT5 by sponging miR-138-5p/miR-432-5p, inhibiting the progression of Glioblastoma. Similarly, Xiang et al. (2020) found that circ-Foxo3 was highly expressed in Gastric cancer cell lines and confirmed the existence of the circ-Foxo3/miR-143-3p/USP44 axis via a series of assays based on bioinformatics analysis Kong et al. (2020) identified that circ-Foxo3, which targets SLC25A15, combined miR-29a-3p to promote the proliferation, invasion, apoptosis and tumorigenesis of prostate cancer. Besides, Yan et al. (2020) demonstrated that circHIPK3, increasing Foxo3a expression, serves as a miR-421 sponge and inhibited inflammation to prevent ischemic injury.

### Interaction Between CircRNAs and Proteins

Similarly, circRNAs can also interact with proteins with their binding sites and regulate the function of proteins (Figure 1B) (Yang et al., 2017). CircRNAs could regulate the expressions and functions of proteins, and proteins could also influence the synthesis and degradation of circRNAs (Huang et al., 2020). For instance, Nair et al. (2016) demonstrated that, as a negative regulator, circ-Foxo3 could promote the degradation of p53 through binding to murine double minute2 (MDM2), leading to an increase in Foxo3 protein expression. Under the dual role of circ-Foxo3, the progression of breast cancer was inhibited via inducing cell apoptosis.

### Translation

A recent study showed that circRNAs, as linear mRNAs, can also be applied as templates for the synthesis of proteins but cannot recruit ribosomes (Jeck et al., 2013). In contrast, circRNAs must be equipped with an open reading frame (ORF) and an internal ribosome entry site (IRES), which is a special nucleotide sequence that is needed for translation of a functional protein or peptide (Figure 1C) (Abe et al., 2015). The article about the translation function of circ-Foxo3 as a template has not been reported yet, while previous study showed that the translation activity of circular template was much lower than the linear counterpart and the modification of circRNAs, especially methylation, is highly correlated with translational activity (Legnini et al., 2017).

### Biomarkers

In recent years, mounting evidence has verified the potential ability of circRNAs to serve as ideal biomarkers for the diagnosis, treatment and prognosis of cancers (Figure 1D) (Deng et al., 2016; Sundaram and Veera Bramhachari, 2017). As shown in previous studies, circRNAs were detected widely in both plasma and exosomes of cell medium (Fan et al., 2019), urine samples (Song et al., 2020), tissues (Zou et al., 2018) and serum (Kun-Peng et al., 2018). Besides, the lack of free ends made circRNAs remarkably stable and resistant to many exonucleases (Suzuki et al., 2006). Importantly, these studies proved that the features of circRNAs, including abundance in tissues or body fluids and stability, could serve as independent biomarkers for cancer diagnosis and prognosis (Huang et al., 2020; Drula et al., 2021).

## Foxo3 in Human Cancers

**FIGURE 2:**
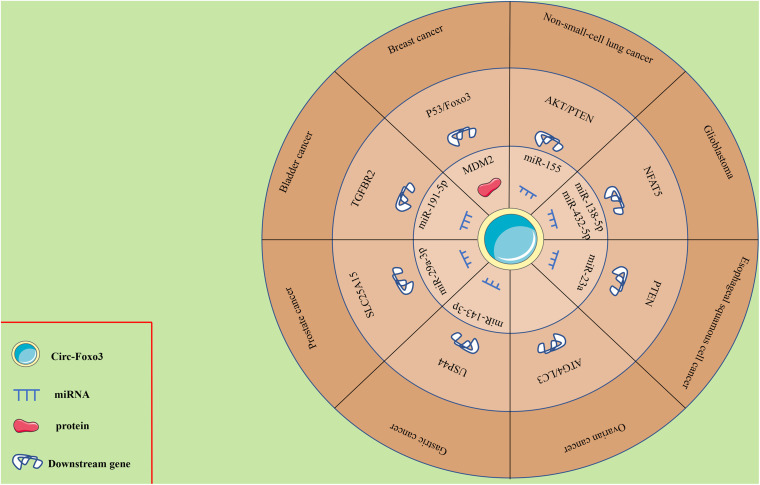
Circ-Foxo3 mediates mechanisms involved in diseases progression.

Mounting evidence has shown the abnormal expression of circ-Foxo3 in numerous human cancers, including breast cancer (BC), lung cancer, glioma, ESCC, leukemia, gastric cancer (GC), bladder cancer, prostate cancer (PCa) and OC. The functional and potential molecular mechanisms of circ-Foxo3 in various human cancers are summarized as follows, and details of the studies are also summarized in Table 1 and Figure 2.

**TABLE 1:** Functional characterization and clinical significance of circ-Foxo3 in human cancers.

Tumor types	Expression	Function role	Clinicopathological features	References
Breast cancer	Downregulation	Suppressing cell proliferation, survival and progression	Better overall survival	Nair et al., 2016; Yang et al., 2016
Non-small cell lung cancer	Downregulation	Suppressing cell proliferation and invasion	Better overall survival and poorer chemoresistance	Myatt and Lam, 2007; Yu et al., 2013; Sun et al., 2014; Zhang Y. et al., 2018
Glioblastoma	Upregulation	Promoting cell proliferation, invasion and tumorigenesis	Advanced tumor size, advanced WHO stage, poorer overall survival, poorer progressive-free survival	Zhang et al., 2019
Acute myeloid leukemia	Downregulation	Not investigated	Better overall survival and poorer chemoresistance	Zhou et al., 2019
Esophageal squamous cell cancer	Downregulation	Suppressing cell proliferation, invasion and migration, arresting cells at the G1 phase and inducing apoptosis	Better overall survival, regressive tumor size	Xing et al., 2020
Ovarian cancer	Downregulation	Inducing apoptosis and autophagy	Better overall survival, regressive tumor size	Lu et al., 2012; Lu et al., 2014
Gastric cancer	Upregulation	Promoting cell proliferation, migration, invasion and tumorigenesis	Poorer overall survival, advanced tumor size	Xiang et al., 2020
Prostate cancer	Downregulation	Suppressing cell proliferation, invasion and inducing apoptosis	Better disease-free survival and poorer chemoresistance	Shen et al., 2020
	Upregulation	Promoting cell proliferation, invasion and tumorigenesis	Advanced gleason score and tumorigenesis	Kong et al., 2020
Bladder cancer	Downregulation	Suppressing cell proliferation, invasion and migration, and inducing apoptosis	Better overall survival	Wang C. et al., 2019; Li et al., 2020

### Breast Cancer

BC is one of the most common cancers and the leading cause of cancer-related death among females worldwide, and the number of cases is still gradually increasing every year (Gong et al., 2017). Although the condition of BC patients can achieve clinical remission to some extent after comprehensive treatment, one-third of them may experience relapse and chemotherapy resistance or, even worse, metastasis (Goel et al., 2016; Kümler et al., 2016). Therefore, it is necessary to identify novel and effective biomarkers for the early diagnosis and treatment of BC patients.

Nair et al. (2016) demonstrated the vital role of circ-Foxo3 in BC genesis and progression. They presented that the ectopic expression of endogenous circ-Foxo3 led to apoptosis and retardation of tumor progression, while silencing circ-Foxo3 had the opposite effect. They also noted that circ-Foxo3 promoted the ubiquitination and degradation of p53 by binding to MDM2, leading to an increase in Foxo3 protein expression. CircRNAs were found to be enriched in normal mammary tissues rather than tumor tissues, and these findings were probably related to the risk of relapse in BC (Yang et al., 2016). Circ-Foxo3 plays dual roles: it acts as a miRNA sponge that binds to MDM2, and it also interacts with p53. This further enhances the function of MDM2 by regulating the ubiquitination of p53, inducing cell apoptosis, which indirectly inhibits the growth of tumors. In conclusion, circ-Foxo3 could be a novel prognostic biomarker and therapeutic target for patients with BC.

### Non-small-cell Lung Cancer

Lung cancer is the most commonly diagnosed cancer worldwide, accounting for 18.4% of cancer-related deaths in 2018 (Bray et al., 2018). Non-small-cell lung cancer (NSCLC), which accounts for approximately 85% of lung cancers, has a poor prognosis in late-stage patients after surgery, chemotherapy and radiotherapy (Torre et al., 2016). Thus, it is important to find effective biomarkers for the early diagnosis and treatment of lung cancer.

The role of circRNAs is barely known in carcinogenesis and progression, particularly in NSCLC. With the properties of highly conserved sequences and stability, circRNAs are ideal biomarkers for the diagnosis and prognosis of cancers (Osaki et al., 2015; Huang Y. S. et al., 2017). In recent study, Zhang Y. et al. (2018) demonstrated that circ-Foxo3, was downregulated in NSCLC cells and tissue samples. With the progression of cancer, the expression of Foxo3 decreased by inducing the activation of Akt and the silencing of PTEN (Myatt and Lam, 2007). *In vitro*, Zhang verified that circ-Foxo3 inhibits the proliferation, migration and invasion of NSCLC cell lines by sponging miR-155, which is the most amplified miRNA in NSCLC. In addition, previous investigations showed that miR-155, as an oncogene in various cancers, is critical in cancer initiation, progression and chemoresistance (Yu et al., 2013; Sun et al., 2014). Circ-Foxo3 was proven to directly interact with miR-155 and negatively regulate the expression of miR-155. Therefore, circ-Foxo3 can potentially serve as a promising biomarker for NSCLC.

### Glioblastoma

Glioblastoma (GBM) is the most aggressive intracranial tumor of the central nervous system, and the median survival is merely 12 months (Ostrom et al., 2015). Its efficient invasive activity has led scholars to focus on the mechanism of GBM cell invasion. Considering its aggressive behavior, it is necessary to seek prospective biomarkers for the treatment of GBM.

Accumulating studies have shown that circ-Foxo3, as a powerful tumor suppressor that sponges certain miRNAs, is downregulated in many cancers (Yang et al., 2016). Zhang et al. (2019) detected the expression of circ-Foxo3 in 48 gliomas and 10 normal samples, and circ-Foxo3 was found to be expressed at a significantly higher level in the GBM group. According to their study, circ-Foxo3 expression was not only significantly associated with tumor size and histologic grade but was also related to wild-type isocitrate dehydrogenase (IDH) expression and O6-methylguanine DNA methyltransferase (MGMT) methylation status. An anti-AGO2 RIP assay was performed in U87-MG cells and showed that circ-Foxo3 and miR-138-5p/miR-432-5p were enriched for AGO2, indicating that circ-Foxo3 binds miR-138-5p/miR-432-5p as a miRNA sponge. Western blot analysis showed that NFAT5 expression was downregulated in cells overexpressing miR-138-5p or miR-432-5p. In contrast, adding a miR-138-5p or miR-432-5p inhibitor increased NFAT5 expression. These findings proved that miR-138-5p/miR-432-5p could bind to circ-Foxo3 and inhibit the proliferation and invasion of GBM cells by targeting NFAT5. Above all, circ-Foxo3 is still considered a novel factor and potential therapeutic target for GBM patients.

### Acute Myeloid Leukemia

Acute myeloid leukemia (AML) is the most common type of hematological neoplasm among people of all ages, and more than 20,000 patients are newly diagnosed in the United States each year (De Kouchkovsky and Abdul-Hay, 2016). The clinical, pathological, molecular and genetic characteristics, which are often heterogeneous, are vital for determining the prognosis of AML patients. Thus, effective biomarkers for AML patients are urgently needed.

Zhou et al. (2019) found that AML patients with high expression of circ-Foxo3 tended to live longer than those with low expression of circ-Foxo3, as detected by Kaplan-Meier analysis. They also observed that patients with high circ-Foxo3 expression usually had longer survival times than those with low circ-Foxo3 expression after chemotherapy and inferred that lower circ-Foxo3 expression was closely related to drug resistance. Although AML-associated miRNAs have not yet been identified, circ-Foxo3 may still be useful in the diagnosis and treatment of AML patients.

### Esophageal Squamous Cell Cancer

ESCC is the major histological type of esophageal cancer (EC) and resulted in approximately 790,000 cancer-associated deaths from 2012 to 2016 (Siegel et al., 2019). Although multiple tools for cancer diagnosis and therapy have developed rapidly, ESCC has high incidence and recurrence rates and is still difficult to detect in the early stages (Huang and Yu, 2018). In addition, the lack of significant change in the overall 5-year survival rate calls for new and sensitive tools for ESCC diagnosis and therapy (Yang et al., 2019).

Xing et al. (2020) observed that circ-Foxo3, play vital physiological roles in ESCC tissues and cell lines via molecular and functional experiments *in vivo* and *in vitro*. Functional assays showed that overexpression of circ-Foxo3 could repress the proliferation and migration of ESCC cells and block these cells at the G1 phase, inducing apoptosis. In addition, miR-23a, which is an oncogene, was upregulated in ESCC cells, leading to cell proliferation (Karimi et al., 2019; Xu et al., 2019; Zhuang et al., 2019). In conclusion, the circ-Foxo3/miR-23a/PTEN signaling pathway highlights some promising therapeutic targets, and the structure of circ-Foxo3 could make circ-Foxo3 a reliable biomarker for ESCC patients.

### Ovarian Cancer

OC is the second most common malignant gynecological tumor, accounting for 5% of all malignant gynecological tumors (Torre et al., 2018). Due to the lack of early clinical symptoms and specific biomarkers, the 5-year survival rate of patients is merely 40%, and these patients often have a high risk of recurrence and metastasis (Su et al., 2013). Thus, seeking effective biomarkers for early diagnosis and treatment would be of great clinical significance for OC patients.

Lu et al. (2014) demonstrated that aplasia Ras homolog member I (ARHI; DIRAS3) increased Foxo3a-mediated expression of Rab7; the formation of functional autolysosomes requires the successful fusion of autophagosomes and lysosomes through the induction of Rab7. In addition, knockdown of Foxo3a reduced ARHI-mediated induction of the ATG4 and LC3 mRNAs. Lu et al. (2012) revealed that low expression of Foxo3a protein combined with high expression of Skp2 was associated with advanced stage and a poor prognosis in OC patients. Consequently, ARHI correlates with LC3, and Foxo3a contributes to the induction of autophagy in OC cells through multiple mechanisms. In other words, Foxo3a is a promising biomarker that is suitable as a diagnostic marker and indirect therapeutic target.

### Gastric Cancer

According to the Global Cancer Statistics 2018 report, GC is the fourth most common type of human cancer worldwide and is especially prevalent in Eastern Asia (Bray et al., 2018). Unfortunately, the overall 5-year survival rate of patients suffering from advanced GC is still less than 5% despite the establishment of advanced diagnostic methods and treatments (Fang et al., 2015; Reddy et al., 2016). Thus, the lack of specificity and sensitivity of molecular biomarkers for diagnosis is an urgent problem that needs to be solved.

In contrast to certain cancers that express low levels of circ-Foxo3, such as ESCC, BC and bladder cancer, Xiang et al. (2020) found that GC cell lines express significantly higher levels of circ-Foxo3. Thereafter, they conducted functional and molecular assays *in vivo* and *in vitro* to confirm the tumorigenic effect of circ-Foxo3 in GC cells. In light of the circRNA-miRNA-mRNA network, bioinformatics analysis revealed that circ-Foxo3 upregulates the expression of USP44, promoting the progression of GC, and sponges miR-143-3p, and the results were validated by RNA-binding protein immunoprecipitation (RIP) assay, dual-luciferase reporter assay, and RNA pull-down assay (Fang et al., 2019; Ouyang et al., 2019; Wang Z. et al., 2019). Above all, circ-Foxo3 is considered a novel and potential therapeutic target in GC patients.

### Prostate Cancer

PCa is the second most common cancer in males worldwide and the fifth leading cause of cancer-related death among American men (Ziaran et al., 2015; Rawla, 2019). With the development of the world, the incidence of PCa is increasing year by year. Although most PCa are indolent tumors and the 5-year overall survival rate can reach 99%, advanced aggressive PCa is prone to metastasis, especially to the pelvic lymph nodes and the sacrum (Athanazio et al., 2017; Schmid-Alliana et al., 2018). Therefore, it is necessary to find therapeutic targets and prognostic biomarkers for patients with aggressive PCa.

Shen et al. (2020) compared the differences in circ-Foxo3 expression in 46 specimens, including 22 low-grade PCa specimens and 24 high-grade PCa specimens and 18 healthy prostate tissue specimens. Real-time PCR showed that high-grade samples expressed significantly lower levels of circ-Foxo3 than low-grade samples and normal tissue samples. Interestingly, silencing the expression of circ-Foxo3 with short-interfering RNA (siRNA) significantly decreased the expression of circ-Foxo3 at the protein level but not at the mRNA level. Li R. et al. (2018) reported that SIRT3 upregulated the expression of Foxo3a, inhibiting epithelial–mesenchymal transition (EMT) in PCa cells by attenuating the Wnt/β-catenin signaling pathway. Similarly, Shen also declared that the silencing of circ-Foxo3 promoted tumorigenesis and resistance to drugs, especially docetaxel, by enhancing EMT. Both the circ-Foxo3/Foxo3/EMT axis and SIRT3/Wnt/β-catenin/Foxo3A are promising prognostic and therapeutic targets for PCa patients. However, a study carried by Kong et al. (2020) showed a converse perspective about the function of circ-Foxo3. Their study found that circ-Foxo3 could promote PCa progression by sponging miR-29a-3p, which targets SLC25A15. Overall, there is no doubt that circ-Foxo3 could be a sensitive biomarker for PCa patients, although the specific expression of circ-Foxo3 and its effects on PCa cells remain to be investigated.

### Bladder Cancer

Bladder cancer, also called urothelial carcinoma (UC), is the most lethal and commonly diagnosed genitourinary cancer worldwide, ranking as the 4th most common cancer in males, and the treatment standard mainly involves surgery, supplemented by radiotherapy and chemotherapy (Antoni et al., 2017; Pal et al., 2019; Crispen and Kusmartsev, 2020). The most common type of UC is transitional cell carcinoma, which originates from urothelial cells, and the potential risk factors of UC, such as smoking and exposure to certain carcinogens, are well known. However, the progression, recurrence and mortality rates of UC patients still remain high. Thus, there is an urgent need to explore effective and stable biomarkers for UC diagnosis and treatment.

Wang C. et al. (2019) demonstrated that circ-Foxo3 targeting miR-191-5p was downregulated in bladder cancer tissue through a series of experiments *in vivo* and *in vitro*. After inducing the apoptosis of bladder cancer cells, they found that the expression of circ-Foxo3 was significantly upregulated in relation to apoptotic stress. Consistently, the overexpression of circ-Foxo3 *in vivo* actually promoted the apoptosis of bladder cancer cell lines. Regarding previous research on miR-191, miR-191-5p has been associated with multiple tumors and has attracted the attention of scholars (Nagpal et al., 2015; Chen et al., 2018). The interaction between circ-Foxo3 and miR-191-5p was examined by luciferase reporter assay, and the results confirmed that miR-191-5p could target circ-Foxo3 to directly suppress the expression of circ-Foxo3. Li et al. (2020) also assessed the expression of circ-Foxo3 in UC cells. In contrast, they focused on the circ-Foxo3/miR-9-5p/TGFBR2 axis and verified via a series of assays that circ-Foxo3 suppressed the proliferation, migration and invasion of UC cells by inhibiting miR-9-5p and promoting the expression of TGFBR2. In summary, circ-Foxo3, as a widely accepted tumor suppressor in UC, is a potential therapeutic target and prognostic biomarker for UC patients.

## Mechanistic Model of Circ-Foxo3 in Human Cancer

Emerging studies have demonstrated that circRNAs, especially circ-Foxo3, could play a role as tumor promoters or tumor suppressors in various human cancers via diverse regulatory mechanisms. To better understand tumorigenesis and circRNA-based therapeutics, a model of the mechanism of circ-Foxo3 in human cancers is necessary.

### The Decoy Mechanism

The special back-spliced junction (BSJ) structure is determined by the binding of cis and trans factors. Trans-acting factors, especially RNA-binding proteins (RBPs), regulate hundreds of circRNA levels through multiple mechanisms (Dong R. et al., 2017; Errichelli et al., 2017; Fei et al., 2017; Li et al., 2020). The decoy mechanism of these factors is considered a mechanism for the negative regulation of miRNA (Hansen et al., 2013). At the protein level, quite high concentrations of circRNAs are required, and circRNAs have different effects on different proteins (Li et al., 2017). For example, Yang et al. (2016) tested the binding of circ-Foxo3, Foxo3P and Foxo3 mRNA with labeled miRNA mimics, including miR-22, miR-136, miR-138, miR-149, miR-433, miR-762, miR-3614-5p, and miR-3622b-5p, by real-time PCR. They found that all miRNA mimics downregulated significantly the expression of circ-Foxo3 in the cells transfected with circ-Foxo3 construct and proved the ability of circ-Foxo3 acting as sponges through binding to miRNAs. Overall, it was confirmed that the miRNA-sponging effect of circRNAs negatively correlates with the level of miRNA. However, the interactions between circRNAs and proteins need to be further investigated.

### Posttranscriptional Regulation

The ceRNA theory indicates that the presence of regulatory networks, especially lncRNA-miRNA-mRNA networks, is closely related to tumorigenesis (Sumazin et al., 2011; Nitzan et al., 2014; Song et al., 2017). Recently, circ-Foxo3 was verified to play critical roles in the ceRNA network of various human cancers. For example, circ-Foxo3 upregulates PTEN expression via the circ-Foxo3/miR-23a/mRNA PTEN and circ-Foxo3/miR-155/mRNA AKT/PTEN axes in ESCC and NSCLC, respectively (Zhang Y. et al., 2018; Karimi et al., 2019). Consistently, circ-Foxo3 suppresses bladder cancer progression through circ-Foxo3/miR-191-5p/mRNA cleaved caspase-3 and circ-Foxo3/miR9-5p/mRNA TGFBR2 (Wang C. et al., 2019; Li et al., 2020). However, circ-Foxo3 exerts a different role in GC, PCa and GBM—it promotes tumor progression via the circ-Foxo3/miR-143-3p/mRNA USP44, circ-Foxo3/miR-29a-3p/mRNA SLC25A15, and circ-Foxo3/miR-138-5p/432-5p/mRNA NFAT5 axes (Zhang et al., 2019; Kong et al., 2020; Xiang et al., 2020).

### Sponge Effect of Protein Stability

Emerging studies have revealed the special structure of circRNAs; thus, circRNAs have a few distinct characteristics, such as stability, preservation and specificity (Zhang et al., 2020). Moreover, circRNAs regulate miRNA-mediated mRNA expression via a sponging effect (Meng et al., 2017; Zhang H. D. et al., 2018). Circ-Foxo3 maintains p53 and MDM2 stability in BC cells by facilitating their ubiquitination and degradation through sponging miRNAs. Consequently, this interaction decreases the expression of p53 and MDM2, leading to upregulation of the Foxo3 protein. Consistently, Foxo3a-mediated transcription is enhanced in relation to the expression of ARHI, inducing the transcription of autophagy-related genes (Fang et al., 2011). Low expression of Foxo3a is associated with advanced tumor-node-metastasis (TNM) stage and a poor prognosis in OC. Thus, the interaction of Foxo3a and ARHI is a potential treatment target and prognostic indicator for OC patients. Importantly, circ-Foxo3 could sponge to the cell cycle associated proteins such as CDK2 and p21, thus arresting cells at the G1 phase (Du et al., 2016). In addition, other cell cycle-associated proteins, including CDK6, p16 and p27, were related to circ-Foxo3 closely. This feature showed the potential of circ-Foxo3 in tumor suppressive activity.

## Concluding Remarks and Future Perspectives

Based on the generalization and summarization above, circRNAs, which are novel and potential biomarkers for cancer diagnosis and prognosis and even potential treatment targets, have become a research hotspot (Barrett and Salzman, 2016; Dong Y. et al., 2017; Li et al., 2017; Zhang H. D. et al., 2018). Nevertheless, there are still areas of circRNA research that need further discussion, such as the identification of circRNAs that lack poly A tails, the mechanisms by which circRNAs promote or inhibit tumor progression and the standardization of the circRNA naming system (Kristensen et al., 2018).

The expression of circ-Foxo3 in various cancers has been tested and compared, and the expression trends of circ-Foxo3 in different cancers are not completely consistent. For example, the expression of circ-Foxo3 in BC (Nair et al., 2016), EC (Yang et al., 2019), lung cancer (Zhang Y. et al., 2018) and bladder cancer (Wang C. et al., 2019) is downregulated, while circ-Foxo3 is usually overexpressed in GC (Sun et al., 2014) and glioma (Zhang et al., 2019) compared with normal controls. However, circ-Foxo3 expression and its specific function in PCa are controversial and remain to be further studied. Nevertheless, it is undisputed that circ-Foxo3 has a significant effect on modulating cell proliferation, migration, invasion and apoptosis through different signaling pathways (Yang et al., 2016). In addition, aberrant expression of circ-Foxo3 is remarkably correlated with certain clinicopathological features of cancer patients, reflecting its potential value as an effective and sensitive biomarker for cancer diagnosis, prognosis and treatment. Moreover, numerous studies have elucidated that circ-Foxo3 could act as a ceRNA with a circular structure to sponge different miRNAs that target upstream modulators, regulating the expression of oncogenes and tumor suppressors (Nitzan et al., 2014). Although previous studies showed that the aberrant expression of circ-Foxo3 was involved in the diagnosis and prognosis of diseases, Lin et al. (2020) focused on the Foxo3/Bim_EL_ pathway activated by circ-Foxo3 which inducing mitochondrial-mediated apoptosis, suggesting a potential target of treatment.

In conclusion, circ-Foxo3 serves as a promising diagnostic and prognostic biomarker in various cancers. Mounting research has focused on studying the biological function and molecular mechanism of circ-Foxo3 in different cancers; thus, the most recent work is still at the molecular level. Consequently, the lack of studies regarding the correlation of circ-Foxo3 with clinicopathological characteristics is a major limitation for its clinical application. In the future, more clinical tissue specimens should be utilized to further study the expression pattern of circ-Foxo3 and the interrelations among circ-Foxo3 level, clinical parameters and prognosis in different cancer patients. Furthermore, studying the expression levels and molecular mechanism of circRNAs in body fluids, which have been previously underinvestigated, will be of great value for the early diagnosis and prognostication of cancers. Overall, we strongly believe that, with further studies, circ-Foxo3 can be utilized in the diagnosis and treatment of patients.

## Author Contributions

TY, YL, and FZ: conception and literature search. TY, LZ, and RJ: manuscript writing and final approval. All authors contributed to the article and approved the submitted version.

## Conflict of Interest

The authors declare that the research was conducted in the absence of any commercial or financial relationships that could be construed as a potential conflict of interest.
